# The Vital Force Defined

**Published:** 1892-03

**Authors:** M. W. Vandenburg


### THE VITAL FORCE DEFINED.

Editor of The Homœopathic Physician:

Allow me to enter my protest to the unfortunate naming of an article in the February number of The Homœopathic Physician, page 63. Had you said Swedenborg's definition of vital force, the suitability of the definition would have been apparent; you might have added, by way of parenthesis, Dr. Bayard indorses this.

Again I must vigorously protest against the imputation that might be drawn from seeing this in a reputable homœopathic journal, that this is Homœopathy, or has the slightest to do with that branch of medicine. Homœopathy has no more to do with the mysticism of Swedenborg than it has to do with the thirty-nine articles of the Episcopalian Church, or the decrees of the Vatican, or the mysticism of the Methodists. Let us leave out our religious views and preferences when we talk Homœopathy, and so avoid discussion on points on which we are sure to disagree.

Neither metaphysics nor religious views have anything to do with the practice of homœopathic medicine. On these points we are at full liberty to hold such views as we please, and we may hold diametrically opposite views on these questions, without in any way impugning our medical faith, or lessening the zeal with which we pursue the same object, the development and application of the law of cure.

M. W. Vandenburg.

